# The Industrial Residue of Andiroba (*Carapa* sp.): A Promising Source of Natural Acaricides Against *Dermacentor nitens* (Acari: Ixodidae)

**DOI:** 10.3390/vetsci12050421

**Published:** 2025-04-29

**Authors:** Daniela Bianchi, Raidel Reis dos Santos, Poliana Leão Peleja, Ana Beatriz Barbosa de Sousa, Marcelo da Silva Evangelista, José Sousa de Almeida Júnior, Lauro Euclides Soares Barata, Waldiney Pires Moraes, Antonio Humberto Hamad Minervino

**Affiliations:** 1 PhD Program Society, Nature and Development, PPGSND, Federal University of Western Pará, UFOPA, Santarém 68040-255, PA, Brazil; dani.bio.bianchi@gmail.com (D.B.); raidelrs@gmail.com (R.R.d.S.); polianalepeleja5@gmail.com (P.L.P.); 2 Laboratory of Animal Health, LARSANA, Federal University of Western Pará, UFOPA, Santarém 68040-255, PA, Brazil; anabeatriz4t@gmail.com (A.B.B.d.S.); marcelo.evangelista240@gmail.com (M.d.S.E.); 3 Laboratório de Farmacologia Experimental, Universidade Federal do Oeste do Pará, UFOPA, Santarém 68040-255, PA, Brazil; jsalmeidajr@hotmail.com (J.S.d.A.J.); lauroesbarata@gmail.com (L.E.S.B.); waldineypires@gmail.com (W.P.M.)

**Keywords:** tropical horse tick, Amazon, natural products, acaricide activity

## Abstract

*Dermacentor nitens* (tropical horse tick) is a tick species commonly found parasitizing horses and other equids in the Amazon region, causing economic losses. Currently, tick control is based on chemical acaricides, usually in a power formulation for topical use. However, its indiscriminate use results in the emergence of resistant tick lineages. Several plant compounds have been tested for their acaricide activity. In the Amazon, several plants are used for oil extraction, resulting in the production of solid residue (waste) that may contain chemical molecules with biological activities. Thus, we aimed to test the in vitro acaricidal potential of the ethanolic extract of the industrial residue of andiroba (*Carapa* sp.) against *D. nitens*. In a larval immersion test (LIT), *D. nitens* larvae were tested with 2.5%, 5%, and 10% andiroba extract concentrations in three replicates. Subsequently, the envelopes were kept in a BOD chamber (28 °C ± 1 °C; 80% ± 5% humidity) for 24 h. At the end of this period, live and dead larvae were counted, and efficiency was calculated from the mortality rate caused by the extract and corrected by the Abbot formula. In the three repetitions, the andiroba extract at a concentration of 5% caused the death of 100% of the larvae. At concentrations of 2.5% and 10%, they caused the death of, respectively, 12.38% (±3.5%) and 69.79% (±7.98%) of the larvae. These results indicate that the extract from the industrial residue from the production of andiroba oil has acaricidal activity. It is concluded that the solid waste from industrial andiroba oil extraction has the potential for the development of natural acaricides against *D. nitens*.

## Simple Summary

Andiroba is an Amazonian seed that is pressed for oil extraction. Andiroba residue is usually discarded, but it may still contain substances with biological value. Here, we tested an extract of this residue to see if it can kill tropical horse ticks. We found that the andiroba extract at a 5% concentration can kill all the ticks in the test used. This Amazon plant waste has potential for the development of a natural acaricide.

## 1. Introduction

Ticks are arthropod ectoparasites that need to feed on blood to complete their development. In Brazil, 70 species are currently known, divided between the families Ixodidae and Argasidae [1]. Some of these species are of great medical and veterinary importance, such as the cattle tick (*Rhipicephalus microplus*), which causes great damage to the livestock industry, and the horse ear tick (*Dermacentor nitens*), which transmits the agents of equine piroplasmosis [2].

*D. nitens* feeds on only one host during the parasitic phase of its life cycle, preferring horses. Infested animals suffer from blood spoliation, the formation of wounds that can lead to secondary infections, and the appearance of myiasis. However, the most worrying aspect of this type of parasitism is the transmission of disease-causing pathogens. Equine piroplasmosis is caused by the protozoa *Babesia caballi* and *Theileria equi*, transmitted mainly through the contaminated saliva of ixodid ticks during feeding, and causes symptoms such as fever, lack of appetite, loss of performance, dehydration, and anemia, which can lead to death [3]. In the Amazon region (Western Pará state), a serological study showed a high rate (>30%) and widespread prevalence of *T. equi* antibodies, with tick infestation on horses positively associated with seroprevalence [4].

Piroplasmosis, a tick-borne disease affecting livestock, is primarily managed by controlling its tick vectors using chemical acaricides. Over the years, various synthetic acaricides and their combinations have emerged as the primary tools employed by livestock producers to combat tick populations [3]. However, this approach has encountered significant challenges, including the development of resistant tick strains, environmental contamination, adverse health effects on host animals, and substantial financial costs. Consequently, there is an urgent need to develop alternative tick control strategies that are effective, safe for both humans and animals, environmentally sustainable, and cost-effective. Natural products present challenges for drug discovery, but in recent years the interest in natural products as drug leads is being revitalized [5].

**Figure 1:**
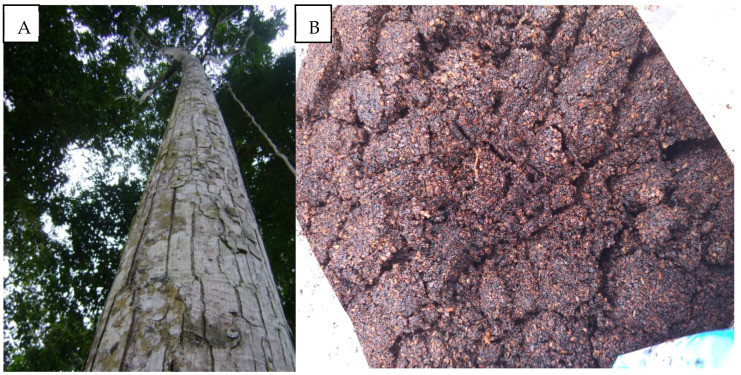
Andiroba *Carapa guianensis* Aublet (Meliaceae) tree (**A**) and the Andiroba seed residue (**B**) after industrialized oil extraction.

Numerous plant-derived compounds have been evaluated for their biological effects on ticks, including repellent activity, decreased tick reproduction, and reduced survival rates across various tick species [6]. These studies often focus on essential oils and secondary metabolites, such as terpenoids and phenolics, which disrupt tick behavior and physiology, offering a promising eco-friendly tick control strategy. In the Amazon region, a diverse array of plant species is exploited for oil extraction, a process that generates substantial quantities of solid waste, including seed husks, pulp, and other by-products, which are typically discarded. Among these, andiroba oil, derived from the seeds of *Carapa guianensis* Aublet (Meliaceae), emerges as a noteworthy example due to its widespread traditional use and considerable production volume [7]. Despite its rich content of bioactive limonoids, which exhibit insect-repellent and anti-inflammatory properties, the residual waste from andiroba oil extraction (Figure 1) remains largely underutilized. Thus, we aimed to test the in vitro acaricidal potential of the ethanolic extract of andiroba industrial waste against *D. nitens*.

## 2. Materials and Methods

Ticks were collected from horses from rural properties in the municipality of Mojuí dos Campos, PA. All animals had no acaricide treatment for at least 30 days before sampling. The engorged females were stored in plastic tubes and taken to the laboratory where they were washed in running water and dried with a paper towel. Then, they were placed in Petri dishes and placed in a BOD-type incubator at a controlled temperature (28 °C ± 1 °C) and humidity (80% ± 5%) until the end of oviposition. Then, aliquots of 0.250 g of eggs were placed in plastic syringes sealed with hydrophilic cotton to ensure the passage of air and moisture while preventing the exit of the larvae after hatching.

The industrial residue of andiroba (*Carapa guianensis*) was supplied by a local company, and the ethanol extract was produced with ethyl alcohol 99.8% P.A. (Neon, Suzano, Brazil) in a Soxhlet-type extractor. For additional information regarding Andiroba oil production systems, please see Supplementary Materials Figures S1–S3. Solutions containing 10%, 5%, and 2.5% of ethanolic extract diluted with 1% Tween 80 were tested. The chemical characterization of the extract was performed using ultra-performance liquid chromatography (UPLC-MS) coupled to diode array detectors and a mass spectrometer.

The acaricidal efficiency of the extract was evaluated through larvae immersion tests (LIT) [8]. About 300 (7 to 15 days-old) larvae were placed in 2 mL plastic tubes containing the solution to be tested and kept under mild agitation for five minutes. Then, approximately 100 larvae were transferred to qualitative filter paper envelopes and sealed with Binder-type clips. We used three envelopes for each extract concentration and three envelopes for each control, totaling 15 LIT. Distilled water and 1% Tween 80 were used as controls. The envelopes were kept in a BOD oven, under the same conditions as the teleogynes, for 24 h. At the end of 24 h, live and dead larvae were counted with the aid of a vacuum pump, and only larvae that could walk were considered alive.

The efficiency of each tested extract concentration was calculated from the mortality rate (MT) of the larvae (1) and corrected by the Abbot Formula (2) [9]. For the corrected mortality formula, we considered the results of the Tween 80 control.

MR = (Dead larvae/total larvae) × 100(1)

Abbot = [(TM treated group − TM control group)/100 − TM control group] × 100(2)

## 3. Results

**Figure 2:**
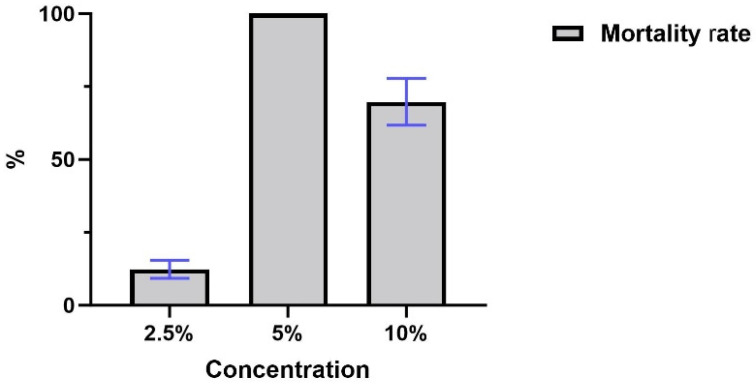
Comparison between mean values and standard deviation of mortality obtained at each concentration. Blue bar indicate standard deviation.

All concentrations of ethanolic extract from the industrial residue of andiroba showed some degree of acaricidal activity. The lowest concentration (2.5%) had a limited larval mortality rate of around 12.38% (±3.5), but the 5% solution showed the most promising results, causing the death of 100% of the larvae in all tests. The 10% solution exhibited intermediate results, causing 69.79% (±7.97) mean larval mortality (Figure 2).

In this study, 40.6% of the compounds present in the extract were identified. The most abundant were gallic acid, gentisinic acid, andirolide S, andirolide B, copalic acid, 6,7-dihydroflavopereirin and geissolaevina. This is a preliminary report dealing specifically with acaricidal activity; the complete chemical characterization of the Andiroba extract used will be published in a separate article.

## 4. Discussion

Unexpectedly, our results did not behave consistently, with a smaller concentration of 5% being more effective than a 10% concentration. These results prevented the calculation of the LC_50_ and LC_95_ of the plant residue extract against *D. nitens*. Unfortunately, we could not secure additional *D. nitens* engorged females in condition to lay eggs in the laboratory for a further repetition of the tests with a new batch of field-collected ticks, especially because farmers have the practice of applying topical acaricide weekly in the equids’ ears, resulting in limited availability of ticks without previous acaricide contact. Some hypotheses can be formulated to explain this unexpected result. The andiroba residue may present solubility and stability issues (i.e., at higher concentrations, the natural product might face solubility or stability issues, reducing its bioavailability). The andiroba residue in a 10% ethanolic extract may present synergistic or antagonistic effects in mixtures; the relative proportions of these compounds at different concentrations could lead to synergistic or antagonistic effects. Natural products have complex compositions that can lead to non-linear effects [10].

The natural product’s mode of action might be optimized at lower concentrations, and higher concentrations might lead to saturation or inhibition of the specific acaricide effect. Non-monotonic dose–response curves are documented in toxicology studies, where low doses can be more effective due to complex mechanisms [11,12].

The andiroba residue extract may exhibit a hormetic dose response (characterized by a low-dose stimulation and a high-dose inhibition) [13]. Further studies with a wide range of concentrations are required to elucidate these results.

The acaricidal action of the oil extracted from the seeds of *C. guianensis* is evidenced in different publications. In a study carried out with engorged females of *D. nitens* [14], andiroba oil at a concentration of 10% caused total inhibition of the hatching of eggs deposited by treated females. Another study evaluating the effects of oil on cells of the reproductive system of a different tick, *Rhipicephalus sanguineus,* demonstrated that at a concentration of 5%, it was possible to observe cytotoxic effects [15]. However, the chemical composition of andiroba oil and the ethanolic extract of the industrial residue of andiroba are different; while the oil is rich in unsaturated fatty acids, such as oleic, palmitic, stearic, and linoleic acids, the ethanolic extract of the residue is composed mainly of terpenoids. Therefore, although both have acaricidal action, the form and intensity of the action are also different.

Although no information regarding *D. nitens* acaricide resistance in the region is available, a previous report showed that other tick species from farms in Santarém and the Lower Amazon region presented resistance to commercially available acaricides [16], reinforcing the need for new acaricide formulations. Botanical insecticides often have lower environmental persistence and impact on non-target organisms [17], and thus a natural acaricide obtained from an Amazonian seed would have limited environmental impact and may contribute to the bioeconomic development of the region. Producing an additional source of income for the local population (from the Andiroba solid waste) will contribute to reduced pressure on forests by offering alternatives to deforestation-driven activities [18]. Further studies evaluating different concentrations and complete chemical characterization are required for a better understanding of which compound is responsible for the activity, as well as the mechanism of action of the Andiroba extract on *D. nitens* larvae.

## 5. Conclusions

These are the first records of the acaricidal potential of a solution obtained from *C. guianensis* industrial residue. The extract derived from the industrial byproduct of andiroba oil production has important acaricidal properties. This suggests that the industrial byproduct may be a viable alternative for the formulation of natural acaricidal agents, opening promising possibilities for the control of *Dermacentor nitens* and other related species.

## Data Availability

The data supporting the results of this study can be found in Supporting Information. Any other information or data could be provided upon request.

## Supplementary Materials

The following supporting information can be downloaded at: https://www.mdpi.com/article/10.3390/vetsci12050421/s1, Figure S1: Area of planted Andiroba trees (A) and a detailed images of the Andiroba seeds (B) and (C). Photos gently given by Dr. Everton Almeida, Dra. Josineide Pamplona, Ms. Mayara Duarte and Mr. Guilherme Sousa; Figure S2: Traditional process for Andiroba oil extraction: Seeds must be sundried covered of the rain. (A) (images from Pajurá indigenous community). In small scale production (B) the seed are cooked with boiling water, then the excess water is removed until obtain an Andiroba paste (C) which is stored with inclination (D) for the Andiroba oil slowly drain, process that can take several days. Photos gently given by Dra. Josineide Pamplona and Mr. Guilherme Sousa; Figure S3: Description of industrial process for Andiroba oil extraction. Seeds after drying were processed by industrial oil presses (A) for extraction of Andiroba oil, producing large amounts of solid residue (B), used in this work as plant material for the production of an ethanolic extract. Photos gently given by Dr. Everton Almeida.
