# Supplementary material for: The Industrial Residue of Andiroba (Carapa sp.): A Promising Source of Natural Acaricides Against Dermacentor nitens (Acari: Ixodidae)
Source: Vet Sci. 2025 Apr 29;12(5):421. doi: 10.3390/vetsci12050421 (PMC12115955; doi:10.3390/vetsci12050421)

Supplementary Material

Figure S1. Area of planted Andiroba trees (A) and a detailed images of the Andiroba seeds (B) and (C). Photos gently given by Dr. Everton Almeida, Dra. Josineide Pamplona, Ms. Mayara Duarte and Mr. Guilherme Sousa.

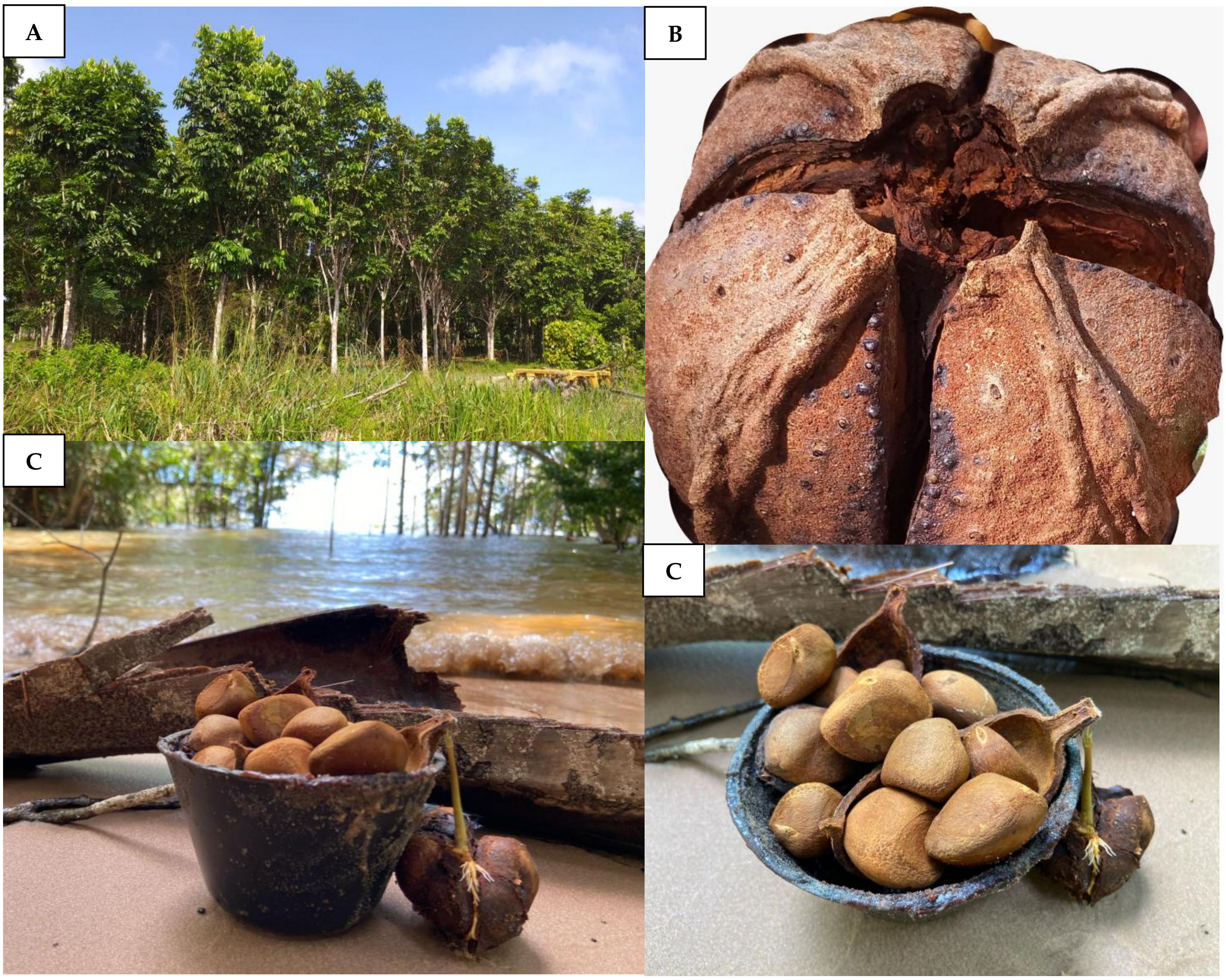

Figure S2. Traditional process for Andiroba oil extraction: Seeds must be sundried covered of the rain. (A) (images from Pajurá indigenous community). In small scale production (B) the seed are cooked with boiling water, then the excess water is removed until obtain an Andiroba paste (C) which is stored with inclination (D) for the Andiroba oil slowly drain, process that can take several days. Photos gently given by Dra. Josineide Pamplona and Mr. Guilherme Sousa.

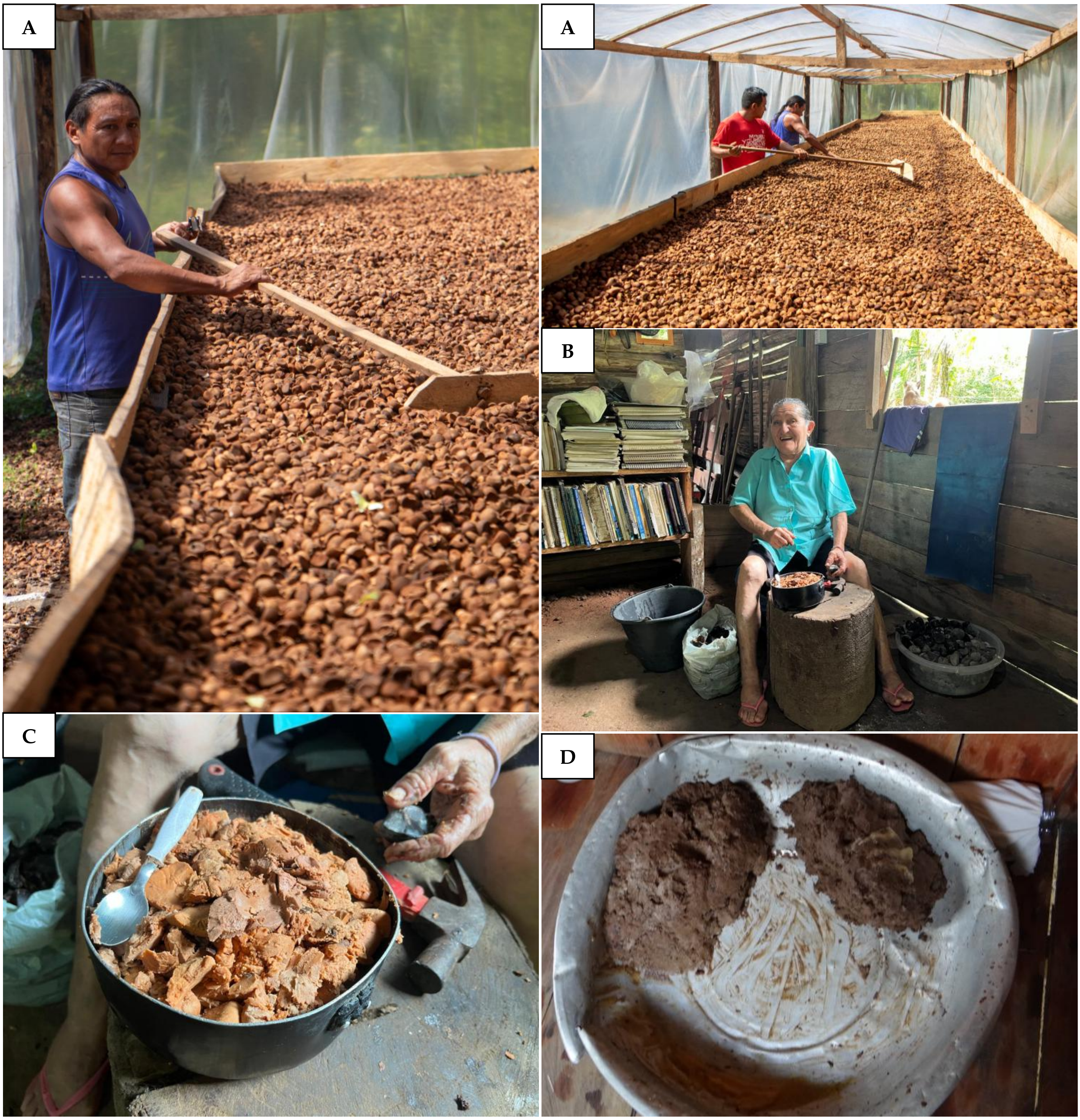

Figure S3. Description of industrial process for Andiroba oil extraction. Seeds after drying were processed by industrial oil presses (A) for extraction of Andiroba oil, producing large amounts of solid residue (B), used in this work as plant material for the production of an ethanolic extract. Photos gently given by Dr. Everton Almeida.

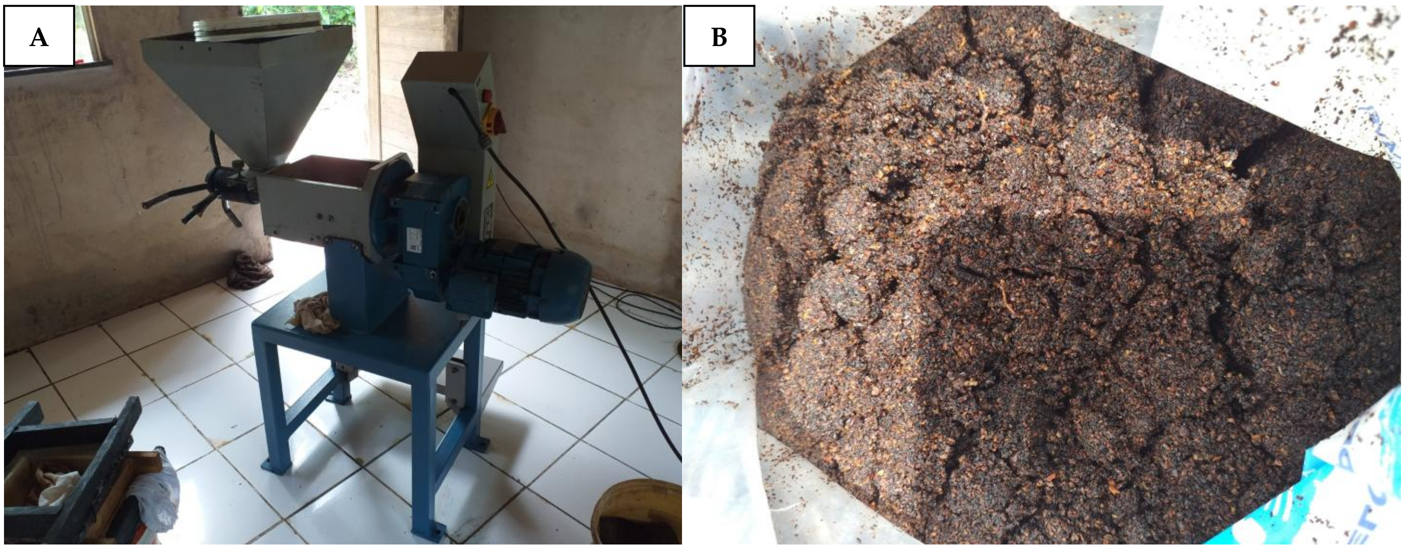

Supplement: Supplementary file 1 [file vetsci-12-00421-s001.zip › vetsci-3554076-supplementary.pdf]
